# Daily consumption of pro-vitamin A biofortified (yellow) cassava improves serum retinol concentrations in preschool children in Nigeria: a randomized controlled trial

**DOI:** 10.1093/ajcn/nqaa290

**Published:** 2020-11-12

**Authors:** Ibukun Afolami, Martin N Mwangi, Folake Samuel, Erick Boy, Paul Ilona, Elise F Talsma, Edith Feskens, Alida Melse-Boonstra

**Affiliations:** Division of Human Nutrition and Health, Wageningen University and Research, Wageningen, Netherlands; Department of Human Nutrition and Dietetics, University of Ibadan, Ibadan, Nigeria; Division of Human Nutrition and Health, Wageningen University and Research, Wageningen, Netherlands; Training and Research Unit of Excellence, College of Medicine, University of Malawi, Blantyre, Malawi; Department of Human Nutrition and Dietetics, University of Ibadan, Ibadan, Nigeria; HarvestPlus, Washington, DC, USA; HarvestPlus, Ibadan, Nigeria; Division of Human Nutrition and Health, Wageningen University and Research, Wageningen, Netherlands; Division of Human Nutrition and Health, Wageningen University and Research, Wageningen, Netherlands; Division of Human Nutrition and Health, Wageningen University and Research, Wageningen, Netherlands

**Keywords:** vitamin A, biofortification, cassava, preschool children, Nigeria

## Abstract

**Background:**

Vitamin A deficiency is a public health problem in sub-Saharan Africa. Pro-vitamin A biofortified (yellow) cassava has the potential to contribute significantly to improve vitamin A status, especially in populations that are difficult to reach with other strategies.

**Objectives:**

The study aimed at determining the efficacy of biofortified cassava to improve vitamin A status of Nigerian preschool children.

**Methods:**

An open-label randomized controlled trial was conducted in southwestern Nigeria. In total, 176 preschool children (aged 3–5 y) were randomized into 2 parallel arms comprising an experimental group (*n* = 88), fed foods prepared from biofortified (yellow) cassava, and a control group (*n* = 88), fed foods prepared from white cassava, twice a day, 6 d a week for 93 d.

**Results:**

A total of 159 children completed the trial (yellow cassava group, *n* = 80; white cassava group, *n* = 79). Children consumed 221 and 74 µg/d retinol activity equivalents from intervention foods in the yellow and white cassava groups, respectively. The treatment effect on serum retinol concentrations at the end of the feeding trial was 0.06 µmol/L (95% CI: 0.004, 0.124 µmol/L), after adjustment for baseline retinol concentrations, inflammation, and asymptomatic malaria status. No significant treatment effects were detected for serum β-carotene (adjusted effect: 3.9%; 95% CI: −0.6%, 8.6%) and gut permeability (adjusted effect: 0.002; 95% CI: −0.089, 0.092), but a significant effect was detected for hemoglobin concentrations (adjusted effect: 3.08 g/L; 95% CI: 0.38, 5.78 g/L).

**Conclusions:**

Daily consumption of β-carotene from biofortified cassava improved serum retinol and hemoglobin concentrations modestly in Nigerian preschool children. This study was registered with clinicaltrials.gov as NCT02627222.

## Introduction

Vitamin A deficiency (VAD) is a global public health problem with high prevalence especially in developing regions of the world. Biofortification is a food-based approach aimed at reducing the global burden of micronutrient deficiencies, including VAD. Currently, orange flesh sweet potato, yellow cassava, and orange maize are being used as biofortified staples to deliver pro-vitamin A across large populations in Uganda, Nigeria, and Zambia, among others (1). Efficacy trials conducted with some of these crops have shown modest to ample effects so far, proving the principle that pro-vitamin A biofortified foods can contribute significantly to total body pools of retinol (2–5).

Nigeria, with an estimated population of 200 million, is ranked as the largest producer and consumer of cassava in the world, producing ∼55 million metric tons annually (6). Despite multiple intervention programs to reduce VAD, ∼30% (10 million) of children aged <5 y in the country are still categorized as vitamin A–deficient based on the latest, yet outdated national survey (7). Cassava can therefore be considered as a suitable food choice for the delivery of pro-vitamin A through biofortification in Nigeria (8).

Estimating the effect of a vitamin A intervention is challenging because serum retinol concentrations do not sensitively reflect changes in status when in the sufficient range (9). Moreover, vitamin A status can be highly confounded by inflammation (10) and malaria (11). The presence of malaria has been reported to lower serum retinol concentration independently of inflammation, although the mechanism of this remains unclear (12, 13). Furthermore, as a result of an increase in vitamin A supplementation coverage and fortification efforts, it has become more difficult to prove the efficacy of a food-based approach such as biofortification, especially in preschool children who are targeted by multiple vitamin A programs. Therefore, the retinol isotope dilution (RID) technique to estimate body retinol pools, which captures vitamin A status over the full status range, has emerged as an alternative method to evaluate treatment effects (14, 15). Several efficacy trials with pro-vitamin A–rich foods have shown the usefulness of the RID technique in that respect (2, 3, 16, 17).

In this study, we determined the efficacy of pro-vitamin A biofortified (yellow) cassava in preschool children living in a malaria-endemic region in Nigeria. Our preplanned primary outcome was the total body pool of retinol using the RID technique. However, despite our efforts to implement this technique with methodological rigor and informed advice from international experts, we were unable to detect the isotope in collected blood samples. Alternatively, we therefore used the difference in change in serum retinol concentration between treatment and control groups as the modified primary outcome of the study. The study was registered with clinicaltrials.gov as NCT02627222.

## Methods

### Study population and subjects

The study was an open-label randomized controlled trial. Study participants were recruited from 3 adjacent rural communities (Telemu, Ashamu, and Ilemowu) in Osun State, southwest Nigeria. These communities were purposively selected based on pre-established criteria, such as rural setting, safety, likelihood of marginal vitamin A status, community goodwill, ease of logistical operations, and proximity to the cassava harvest site. The study area belonged to the rain forest agroecological zone with 2 rainy seasons from March to July and from September to October. The annual rainfall in the country is ∼1165 mm, with a mean annual temperature of 27°C (18). The last national survey in 2004 showed a prevalence of VAD of 22.4% in the study area, which was the highest in the region (7). VAD prevalence is highest in the northern area of Nigeria; however, we could not conduct the study in this location due to concerns regarding the safety of staff and lack of study resources. In the southwest, there is a high *Plasmodium* species entomological inoculation rate of 110 infective bites/person/y (19, 20). Osun State has ∼4009,800 people, predominantly from the Yoruba tribe, who are mostly subsistence farmers growing cassava, yam, and maize (21).

The study was conducted between December 2015 and May 2016. Ethical approval in Nigeria was obtained from the University of Ibadan/University College Hospital Ethics Committee (UI/EC/14/0426). State government approval was obtained from the Ministry of Health in Osun State (OSHREC/PRS/569T/53), as well as positive advice from the Medical Research Ethics Committee of Wageningen University, the Netherlands (14/37). Study participation was on a voluntary basis, and confidentiality of all the obtained information of the participants was maintained throughout the study. Written informed consent, and assent on behalf of the child, was obtained twice from each participant's parent(s) or guardian(s): before the screening and before study commencement.

**FIGURE 1:**
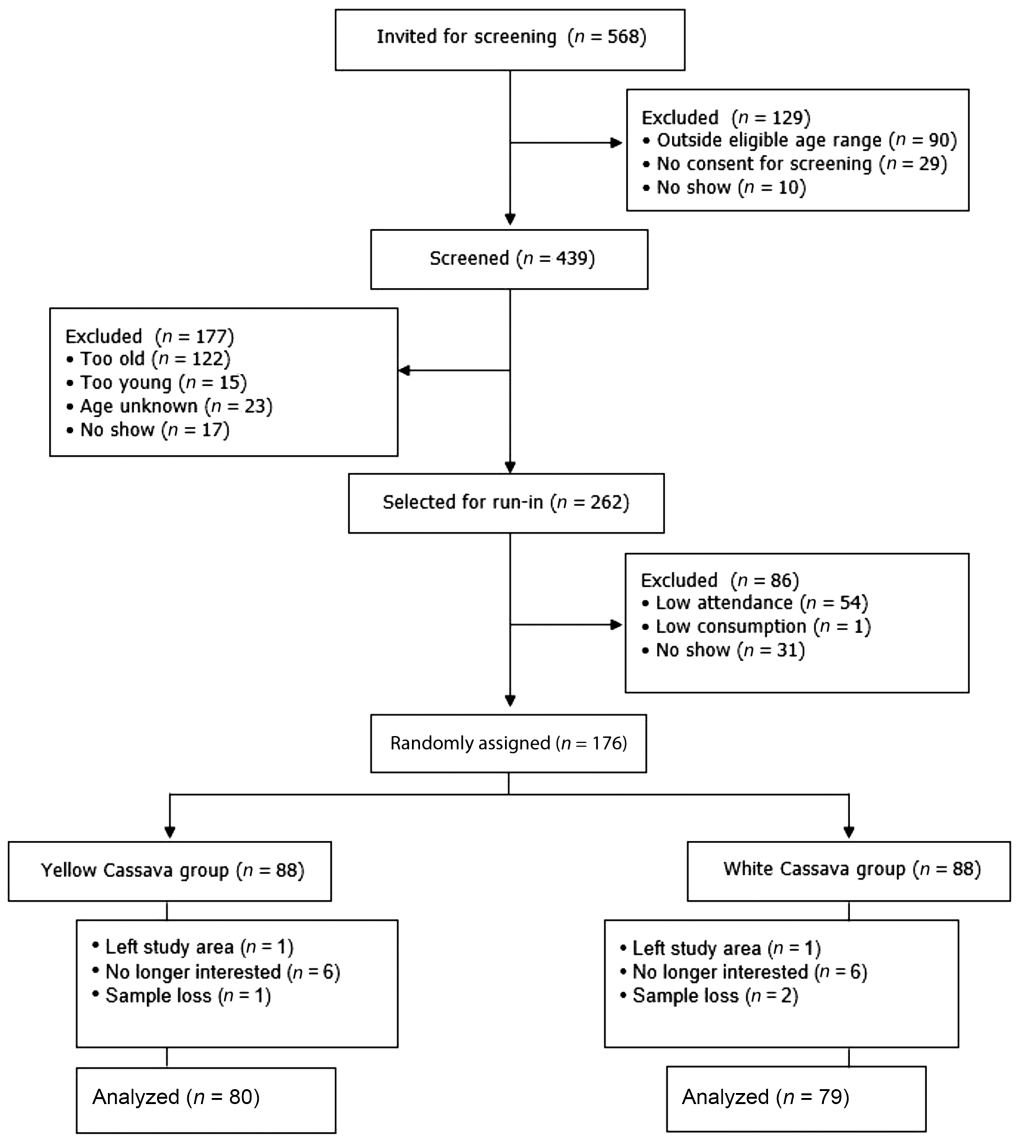
Study flow diagram.

All eligible children in the communities (*n* = 568) were invited to participate (**Figure 1**). Children were screened for eligibility using the following inclusion criteria: *1*) willingness to participate, *2*) no visible sign of sickness, *3*) age >3 y and <5 y before study commencement, and *4*) plasma hemoglobin (Hb) >70 g/L. Serum retinol-binding protein (RBP) was also measured during the screening phase of the study to enable stratified treatment allocation. Most of the children were not enrolled in any school at the beginning of the study. We set up a preschool specifically for the study. A total of 262 screened participants then entered a run-in phase and received a daily ration of control (white) cassava during this period. Children who were frequently absent or were unable to consume ≥80% of the age-specific target meal portion were excluded. During the run-in phase, all children received prophylactic treatment for malaria (lumefantrine/artemether), common intestinal helminth infection (albendazole, 300 mg), and schistosomiasis (praziquantel, 300 mg). Medications were administered ∼2 wk before baseline measurements to eliminate potential factors that could bias vitamin A status estimates at baseline. A total of 176 preschool children, aged 3–5 y, were finally enrolled in the study.

The Nigeria national vitamin A supplementation program has a wide coverage. There was the possibility that recruited children might have just received vitamin A supplements from the national program, which would have introduced possible confounding in our study. We therefore monitored the July 2015 supplementation program in the participating communities and kept a record of all supplemented children within the study location. A period of 4 mo elapsed between the date of the last vitamin A supplementation and commencement of the intervention. The intervention also ended before the next round of supplementation. As a result, we hypothesized that the intervention would prevent a decline in body retinol pools to a certain extent over time, rather than increase body retinol pools.

### Study design and procedures

**FIGURE 2:**
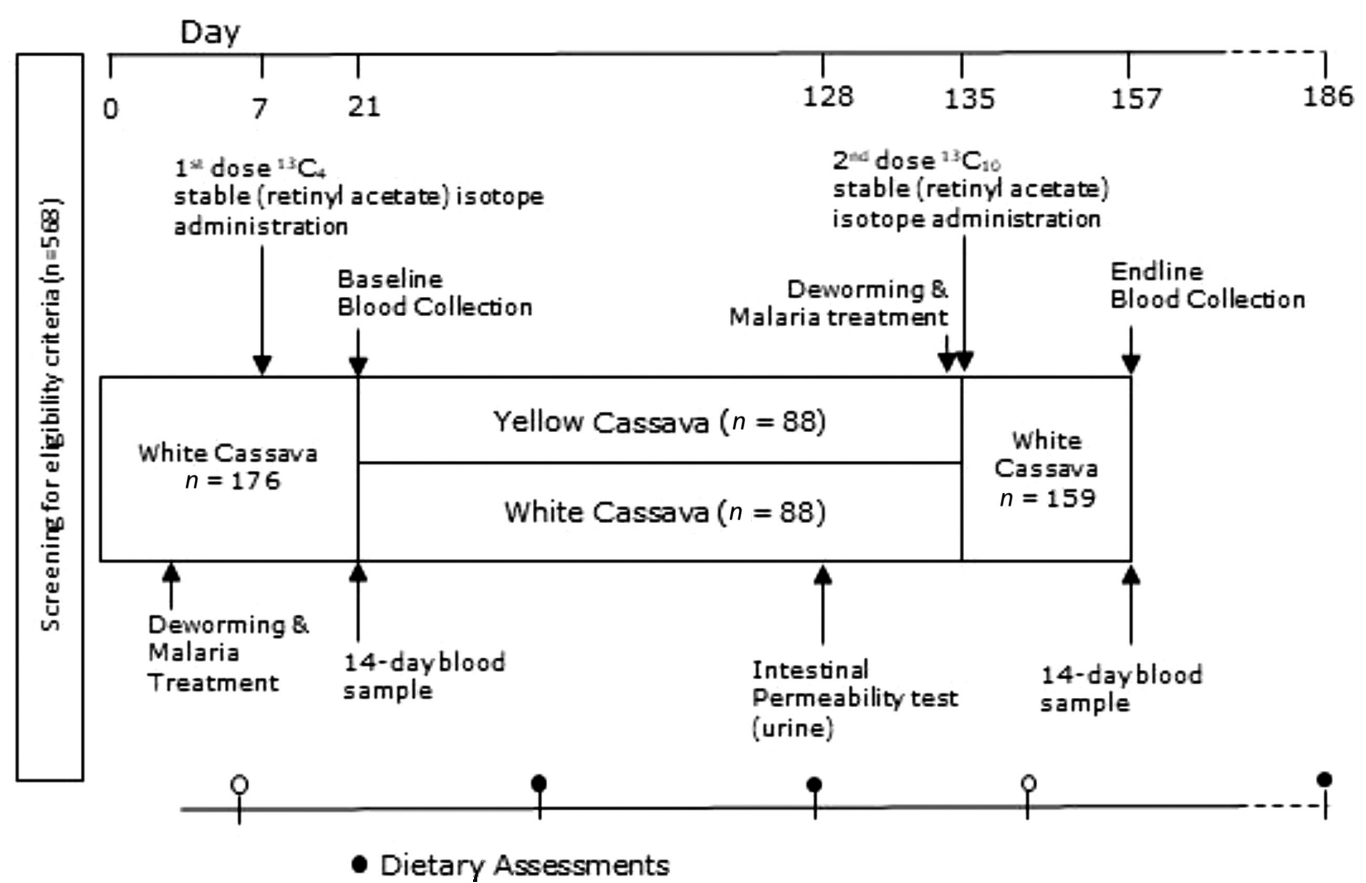
Study design.

The study comprised 2 parallel groups: the treatment and control groups. In the treatment group (*n* = 88), children were fed foods prepared with yellow cassava; in the control group (*n* = 88), children were fed the same menus but the foods were prepared with white cassava. Both the white and the biofortified (yellow) cassava were grown under the supervision of HarvestPlus/International Institute for Tropical Agriculture in Ibadan, Nigeria. The varieties of cassava used in the study were TMS 419 (white cassava) and TMS 07/0593 (yellow cassava). The TMS 07/0593 variety was released in Nigeria in 2013 and was shown to contain a concentration of ∼9 µg/g β-carotene at maturity. Fresh cassava roots were harvested and transported to the research site twice per week. Trained cooks prepared the cassava daily according to standardized recipes (see **Supplemental Methods**) and under supervision with either yellow or white cassava: moinmoin and garri for breakfast and cassava eba with okra or ewedu vegetable soup and stew (with meat) for lunch. Both groups were fed twice a day, breakfast and lunch, 6 d per week, for a total of 16 wk (**Figure 2**).

The preplanned primary outcome of the study was the difference in total body retinol pool between the yellow and white cassava groups to be established by the RID technique. On day 7 (Figure 2), all participants were administered 0.4 mg of stable ^13^C_4_-retinyl acetate tracer, which had been formulated into capsules before the commencement of the study. This was followed by a 14-d equilibration period to enable the labeled tracer to mix with the existing unlabeled body retinol pool (tracee) as applied in other pediatric populations (2, 22). Baseline blood was then collected on day 21, following an overnight fast. At this point, children who successfully passed the run-in phase, and met the eligibility criteria, were randomly assigned using a stratified-block design. Because initial body retinol pools are strong determinants of the response in the study outcome, participants were blocked based on their serum RBP concentration, determined during the screening phase of the study. For this, children were ranked according to their serum RBP concentrations and divided into tertiles of low, medium, and high RBP values.

Subsequently, blocks of 4 and 6 were used to randomize children to the 2 intervention groups within each RBP stratum. Randomization was performed by an independent member of the team, who was not involved in the fieldwork. Because it was practically impossible to blind participants and research assistants to either the treatment (yellow cassava) or the control (white cassava), the study was carried out as an open-label trial.

After baseline measurements and randomization, feeding commenced according to intervention allocation. Feeding locations for the 2 groups were physically separated from each other to prevent crossover between groups. During feeding sessions, the foods were weighed by research assistants into serving containers with known weights and then served immediately to participants. Feeding was constantly monitored by field assistants, and weights of all leftovers were recorded. On day 140 (Figure 2), 0.4 mg of stable ^13^C_10_-retinyl acetate tracer was administered, followed by another 14-d tracer/tracee equilibration. Endline blood samples were collected between days 154 and 157.

We pre-estimated an initial pool size of 12,600 µg (= 44 µmol) retinol; a high-dose vitamin A supplementation of 60,000 µg, 4 mo before baseline; dietary vitamin A intake of 150 µg retinol activity equivalent (RAE) per day; vitamin A absorption/retention of the labeled dose of 0.76; and a fractional catabolic rate of 1.85%/d. Assuming a conservative retinol:β-carotene conversion factor of 1:7 (23), we estimated 150 µg RAE/d as the minimum amount to be consumed by every child in the intervention. Based on these assumptions, we estimated a difference in total body retinol pool size of 4224 µg between groups following intervention, with an SD of 8594 µg, α = 0.05, and 80% power. Assuming a dropout rate of 15%, we estimated a required sample size of *n* = 81 in each study group.

### Preparation of stable isotope

A stock solution of ^13^C_4_ or ^13^C_10_-retinyl acetate (Cambridge Isotope Laboratories), verified for purity and isotopic composition by an external laboratory, was prepared by dissolving 120 mg of each compound in 25 mL ethanol in a volumetric flask. Subsequently, α-tocopherol (∼2 µg/mg retinyl acetate) was added as an antioxidant to the stock solution, and the ethanol was evaporated in the dark under nitrogen gas. Hozol oil (46.68 g) was then added to the residue to make a ^13^C-retinyl acetate concentration of 2.36 mg/mL. Subsequently, 0.17-mL doses were pipetted into opaque gelatin capsules (CapsuGel). Capsules were prepared at the Division of Human Nutrition and Health, Wageningen University, the Netherlands, and stored at 4°C until transportation to the study site and subsequent administration to participants under strict supervision. The amount of retinyl acetate solution in the capsules was verified for each capsule based on capsule weight. Eight capsules were analyzed in duplicate by HPLC and shown to contain 0.37 ± 8 mg of retinyl acetate. Another set of 10 capsules was analyzed after having been transported to the study location, stored in the field during the intervention, and return transported to Europe. These capsules were analyzed by LC-MS/MS in the Lietz’ laboratory at the University of Newcastle upon Tyne, United Kingdom, and contained 0.39 mg of ^13^C_10_-retinyl acetate.

### 24-h recall data collection

In the last week of the intervention, the mean intake of vitamin A and energy was estimated in 162 children from foods consumed within and outside the preschool facility, using the multiple-pass quantitative 24-h dietary recall method (24). Repeated recalls were conducted on nonconsecutive days for 30% of the children to account for intraindividual variation.

### Blood collection, processing, and biochemical assessments

Whole-blood samples were collected by venipuncture into heparinized vacutainers (Becton Dickinson) by phlebotomists from the University College Hospital, Ibadan, Nigeria. Vacutainers were stored immediately in dark containers on ice and were transferred to the field laboratory located next to the preschool for further cooling, centrifugation, aliquoting, and field-specific analysis under yellow light illumination conditions. All samples were processed and stored in liquid nitrogen at −196°C within 12 h of collection until shipment. Samples were shipped frozen on dry ice to Wageningen University, where they were stored at −80°C until analysis.

Concentrations of C-reactive protein (CRP) and Hb (20 µL whole-blood aliquot) were measured in the field at screening, baseline, and endline by the immunoturbidimetric method using a QuickRead-Go CRP/Hb instrument (Orion Diagnostica). Field analyses of CRP and Hb were conducted to immediately identify and treat critical cases of sick children. The instrument was calibrated with Hb and CRP controls obtained from the manufacturer. Plasma concentrations of RBP, ferritin, soluble transferrin receptor, CRP, and α_1_-acid glycoprotein (AGP) were simultaneously determined using a combined simple sandwich ELISA technique in the VitMin laboratory, Wilstaett, Germany (25).

### Small intestine permeability test

Toward the end of the study (Figure 2), small intestine permeability was assessed in participants (*n* = 155) using a multiple sugar gut permeability test (26). For this, 40 g each of lactulose and sucrose and 20 g each of mannitol and rhamnose were weighed into a clean measuring beaker, 1 L of potable water was added, and the sugars were stirred to dissolve. After voiding, each child was administered a 25-mL portion containing 1.0 g each of lactulose and sucrose and 0.5 g each of rhamnose and mannitol. Subsequently, 100–200 mL of potable water was administered to induce urination. Urine was collected during 90 min after sugar dosage, and the time interval between sugar dosage and urine collection was noted. Urine weight and specific gravity were measured immediately after collection using a weighing scale (D-72,336; Kern & Sohn) and urinalysis strip (Surescreen Diagnostics), respectively. Sample aliquots (5 mL) were stored at −20°C between the time of collection and analysis. Urinary lactulose, mannitol, sucrose, and rhamnose were measured by GC using the method described by Jansen et al. (27).

### Determination of serum retinol concentrations

Serum retinol concentration was measured by HPLC (Thermo Scientific Accella LC system; Thermo Fisher Scientific), whereas ^13^C-retinol isotope ratios were determined by LC-MS/MS at the Division of Human Nutrition and Health, Wageningen University, based on the method described by Oxley et al. (28). Both baseline and endline serum samples were analyzed simultaneously to reduce interassay variation. Samples were thawed, homogenized, and pipetted into Kimax tubes. Subsequently, 0.5 mL serum, 0.9% physiologic salt (NaCl; 0.9 w:v% in water), and 1000 µL ethanol (with added retinyl acetate as an internal standard) were added to the samples and extracted twice with 2 mL hexane. The hexane layers were pooled and aerated to dryness under nitrogen using a freeze drier. The extracted residue was dissolved in 125 µL of methanol-1-butanol (for HPLC analysis) or ethyl acetate (for LC-MS analysis), vortexed, and transferred into HPLC vials for analysis. All sample preparation was conducted under subdued yellow light. Retinol and β-carotene were separated on a C18 reversed-phase column (Vydac 201TP52; Grace) using a gradient elution technique. Analytes were detected at 325 nm (retinol and retinyl acetate) and 450 nm (carotenoids) on a photodiode array with multiple wavelength detector. HPLC quantitation was performed using the EZChrom Elite version 3.2.2 SP2 software (Agilent Technologies). Unfortunately, we were not able to detect clear peaks in isotope-enriched retinol in serum samples analyzed by LC-MS/MS, either at baseline or at endline. This was confirmed in the Lietz’ laboratory at the University of Newcastle upon Tyne. Therefore, we could not estimate the total body retinol pools of the children.

### Determination of carotenoids in food

Samples of each recipe, as consumed, were collected randomly every week during the intervention period. Each meal was collected in opaque containers and homogenized using a kitchen blender. Five milliliters of butylhydroquinone (BHT) was added as a preservative to the samples during homogenization. All food samples were stored in opaque plastic jars at −20°C at the field site. After the intervention, samples were shipped to Wageningen University and stored at −80°C until analysis. At the point of analysis, the different food samples were pooled together into composite samples representing 3 stages of the intervention to detect any existing variation in the carotenoid content of the meals during the intervention period. Carotenoids in the foods were analyzed using the same HPLC system described for serum samples. To extract the carotenoids from the foods, we mixed 2 g homogenized food sample, 0.2 g magnesium carbonate, 5 mL deionized water, and 1000 mL ethanol (with added retinyl acetate as internal standard) and extracted 3 times with 20 mL methanoltetrahydrofuran (1:1 vol:vol%) using a rod mixer (Polytron PT 20 OD; Kriens/Luzern) until the residue was colorless. Extracts were filtered on a glass funnel with filter paper (Whatman grade 1; GE Healthcare Life Sciences); the combined filtrates were transferred to a 50-mL volumetric flask and made up to volume with methanoltetrahydrofuran (1:1 vol:vol%). Then, 4 mL filtrate with 1 mL 10% NaCl solution was transferred to a 10-mL glass-stoppered centrifuge tube (Kimax; Kimble Chase), and carotenoids were extracted 3 times with 1.5 mL petroleum ether containing 0.01% butylated hydroxytoluene. The combined ether fractions were evaporated under nitrogen at 358°C. The residue was dissolved in 2 mL methanol/butanol (60/40 vol:vol%), and 1 mL was injected into the HPLC system. Carotenoids were separated on a Vydac 201TP52 column by gradient elution and monitored at 450 nm on a photodiode array detector. Runtime was 20 min per sample. We measured the summed content of *trans* and *cis* β-carotene, the predominant pro-vitamin A carotenoid in yellow cassava (**Supplemental Table 1**).

### Malaria assessment

Malaria infection was measured by rapid dipstick tests based on the detection of histidine-rich protein-2 and *Plasmodium-*specific lactate dehydrogenase (CareStart, product code G0121; Access Bio). In addition, *Plasmodium falciparum*-specific DNA was extracted from erythrocytes, and malaria parasite counts were detected at the Amsterdam Medical Centre, the Netherlands, based on a published protocol (29).

### Anthropometry

Height and weight were measured at baseline and endline using a combined anthropometric scale and stadiometer (model no. 887 7,021,094; serial no. 5877200147452; Seca). Childrens’ ages were verified for 48% of participants by asking each mother/guardian to present the child's birth certificate or immunization card. Verbal recalls were obtained from parents who were unable to provide a formal proof of their child's age. Anthropometric data were analyzed with Anthro (version 3.2.2; WHO), and height-for-age, weight-for-age, and weight-for-height *z* scores were computed.

### Definitions and data analysis

Data analysis was conducted using STATA software (version 13; StataCorp). Nonnormal variables such as CRP and serum β-carotene concentration were log transformed. Serum CRP and AGP are commonly used to adjust for the effect of inflammation on serum retinol concentrations. However, it was recently shown that in a malaria-endemic population, CRP alone explained the highest percentage of the variance in serum retinol concentrations, in addition to the observation that AGP explained little of the remaining variance (30). We thus used only CRP for inflammation adjustment as described by Palmer et al. (30). VAD was defined as serum retinol concentration <0.7 µmol/L, after adjustment for CRP (31). Moderate inflammation was defined as serum CRP >5 mg/L and ≤15 mg/L; high inflammation, serum CRP >15 mg/L (30); anemia, serum Hb <110 g/L; iron deficiency, serum ferritin concentration <30 µg/L when children with inflammation were included or <12 µg/L when children with inflammation were excluded (32); serum soluble transferrin receptor concentration >8.3 µg/mL (33); malaria infection, *Plasmodium* parasite count per microliter of whole blood >0; and stunting, wasting, and underweight were defined as ≤−2 *z* scores of WHO child growth references for height-for-age, weight-for-height, and weight-for-age, respectively (34). Feeding compliance was predefined as the ability to consume the minimum targeted amount of intervention food per day—that is, ≥100 g of eba, ≥32 g of garri, and ≥15 g of moinmoin. Adequate vitamin A intake was defined as 210 µg for children aged <4 y and 275 µg for children aged ≥4 y (35).

ANCOVA was conducted to calculate the unbiased estimate of the average treatment effect (36). In the crude analysis of the primary outcome (serum retinol), an adjustment was made only for randomization stratum, whereas in the adjusted analysis, inflammation-adjusted baseline serum retinol concentration, CRP at endline, and the presence of malaria parasites were included as covariates to adjust for the independent effects of inflammation and malaria on serum retinol concentration. Crude and adjusted models were built for other outcomes similarly by including appropriate variables.

## Results

Out of the 568 participants invited for the study, 77% (*n* = 439) were screened, 46% (*n* = 262) were selected for the run-in, and 31% (*n* = 176) were eventually randomized, after meeting the baseline inclusion criteria (Figure 1). The study recorded a dropout rate of 8% (*n* = 14), mainly because participants’ parents lost interest in the study. In total, data from 159 subjects were analyzed: *n* = 80 samples from the yellow cassava group, and *n* = 79 samples from the white cassava group. At baseline, 23% (*n* = 39) of the participants were stunted, 2% (*n* = 4) wasted, and 13% (*n* = 21) underweight (**Table 1**). Inflammation biomarkers at baseline showed that 25% (*n* = 44) of the children had elevated CRP (>5 mg/L) concentrations, and 5% (*n* = 8) were infected with *P. falciparum*. However, none of the children presented with fever. After correction for inflammation, mean serum retinol concentration was 1.06 µmol/L, and 9% (*n* = 16) of the children were vitamin A deficient at baseline. The mean baseline serum retinol concentration (± SD) of children who had received vitamin A supplementation 4 mo before the study was 0.95 ± 0.25 µmol/L, whereas children who did not receive vitamin A supplementation prior to the study had a mean serum retinol of 1.03 ± 0.02 µmol/L (*P* = 0.16). Iron deficiency was not found in the study population.

**TABLE 1:** Baseline characteristics by intervention group^1^

Characteristic	White cassava group (*n* = 88)	Yellow cassava group (*n* = 88)	*P* value^2^
Age, mo^3^	49.2 ± 10.9^4^	49.1 ± 12.4	0.95
Boys, *n* (%)	51 (58)	49 (56)	0.85
Girls, *n* (%)	37 (42)	39 (44)	
Weight, kg	14.5 ± 1.97	14.4 ± 2.10	0.99
Height, cm	98.2 ± 7.50	98.4 ± 7.43	0.74
WHZ^5^	−0.35 ± 0.84	−0.51 ± 0.82	0.24
WAZ^5^	−1.08 ± 0.98	−1.05 ± 0.94	0.77
HAZ2^5^	−1.36 ± 1.32	−1.18 ± 1.29	0.44
HAZ ≤−2, *n* (%)	23 (28)	16 (18)	0.93
Serum inflammation markers			
AGP, g/L	0.9 (0.6, 1.4)^6^	0.9 (0.6, 1.5)	0.65
CRP, mg/L	1.5 (0.5, 5.1)	0.9 (0.4,5.1)	0.61
CRP <5 mg/L, *n* (%)	66 (75)	66 (75)	0.16
Moderate inflammation, *n* (%)^7^	14 (16)	11 (13)	0.83
High inflammation, *n* (%)^8^	8 (9)	11 (12.5)	0.80
CRP >5 mg/L or AGP >1 g/L, *n* (%)	40 (45.5)	38 (43.2)	0.88
Malaria infection, *n* (%)	1 (1)	7 (8)	
Serum vitamin A markers (all children)			
Retinol, µmol/L	1.08 ± 0.26	1.04 ± 0.26	0.27
Vitamin A deficiency (retinol <0.70 µmol/L), *n* (%)	7 (8)	9 (10)	0.18
β-carotene, µmol/L	1.82 (1.25, 2.48)	1.90 (1.35, 2.42)	0.21
RBP, µmol/L	0.96 (0.80, 1.15)	0.94 (0.78, 1.15)	0.76
Iron markers			
Hb, g/L	116.9 ± 11.5	117.4 ± 15.6	0.90
Anemia, *n* (%)^9^	23 (12.5)	29 (16.5)	0.47
Serum ferritin (all children), µg/L	86.4 ± 34.2	92.8 ± 36.4	0.39
Iron deficiency, *n* (%)^10^	0 (0)	0 (0)	—
Serum ferritin (children without inflammation)	83.5 ± 28.7	79.2 ± 26.7	0.24
Iron deficiency, *n* (%)^10^	0 (0)	0 (0)	—
Serum soluble transferrin receptor, mg/L^10^	7.9 (7.3, 8.5)	8.7 (7.9, 9.4)	0.10

1 AGP, α_1_-acid glycoprotein; CRP, C-reactive protein; HAZ, height-for-age *z* score; Hb, hemoglobin; RBP, retinol-binding protein; WAZ, weight-for-age *z* score; WHZ, weight-for-height *z* score.

2 Statistical significance between groups was calculated using independent *t* test.

3 Age was available for only 82 children in the yellow cassava group and 86 children in the white cassava group.

4 Mean ± SD (all such values).

5 WHZ, WAZ, and HAZ: in yellow cassava group, *n* = 82; in white cassava group, *n* = 86.

6 Median; 25th and 75th percentile in parentheses (all such values).

7 Moderate inflammation is defined as CRP concentrations >5 mg/L and ≤15 mg/L.

8 High inflammation is defined as serum CRP >15 mg/L.

9 Anemia is defined as Hb concentration <110 g/L.

10 Iron deficiency is defined as serum ferritin concentration <30 µg/L when children with inflammation were included or <12 µg/L when children with inflammation were excluded and soluble transferrin receptor concentration >8.3 mg/L.

Participants consumed on average 237 g of eba, 32 g of garri, 50 g of moinmoin, and 65 g of vegetable soup daily in the yellow cassava group and 246 g of eba, 35 g of garri, 49 g of moinmoin, and 74 g of vegetable soup daily in the white cassava group (**Table 2**), with an overall compliance of 98% in both groups. Children attended 87% of the intervention days, with no differences between white cassava and yellow cassava groups; however, a small proportion of children (7%) attended <80% of intervention days. Within the preschool, 221 µg RAE was consumed in the yellow cassava group and 74 µg RAE in the white cassava group per day (Table 2). The median usual RAE intake from foods consumed both in the preschool and at home was 536 µg in the yellow cassava group and 301 µg in the white cassava group (children aged <4 y: 563 µg RAE and 293 µg RAE in the yellow and white cassava groups, respectively; children aged ≥4 y: 469 µg RAE and 303 µg RAE in the yellow and white cassava groups, respectively). The children in the treatment group derived ∼52% of their daily RAE intake from home and 48% from the meal provided by the study. Overall, 37.4% of the daily RAE intake came from the yellow cassava. Children in the control group consumed ∼64% of the amount of daily RAE in comparison with their peers in the treatment group, of which ∼75% was derived from foods at home. In addition, inadequacy of RAE intake was 9% and 29% in the yellow cassava and white cassava groups, respectively. Of the foods consumed at home, palm oil contributed ∼24% and 21% of RAE intake in the yellow and white cassava groups, respectively (*P* = 0.6). There was no statistically significant difference in the adjusted mean RAE intake from foods consumed outside the pre-school between the yellow cassava and white cassava groups (*P* = 0.456).

**TABLE 2:** Estimated daily intake of β-carotene and RAE from intervention foods during the feeding trial^1^

	White cassava group (*n* = 79)			Yellow cassava group (*n* = 80)		
	g/d	β-carotene, µg/d	RAE, µg/d	g/d	β-carotene, µg/d	RAE, µg/d
Cassava-based foods						
Eba	246 (235, 256)	36.9 (35.3, 38.4)	5.3 (5.0, 5.5)	237 (228, 246)	760.8 (731.9, 789.7)	108.57 (104.4, 112.8)
Garri	35 (33, 37)	18.9 (17.2, 20.15)	2.7 (2.5, 2.9)	32 (29, 34)	305.3 (276.7, 324.4)	43.6 (39.5, 46.3)
Moinmoin	49 (45, 53)	66.0 (60.3, 71.0)	10.2 (8.6, 10.1)	50 (45, 54)	133.0 (119.7, 143.6)	19.0 (17.1, 20.5)
Vegetable soups^2^						
Okra	62 (55, 69)	251.4 (224.4, 278.4)	35.9 (32.1, 39.8)	55 (50, 59)	221.1 (152.8, 239.0)	31.6 (21.8, 34.1)
Ewedu	12 (10, 13)	145.5 (127.4, 161.5)	20.8 (18.2, 23.1)	10 (9, 11)	125.2 (115.4, 133.4)	17.9 (16.5, 19.1)
*Total*	—	518.7	74.1	—	1545.4	220.8

1 Values are median (IQR). RAE was calculated based on a β-carotene:retinol conversion factor of 7:1 (23). RAE, retinol activity equivalent.

2 The vegetable soup was a mixture of cooked okra + stew and cooked Ewedu + stew (see Supplemental Methods for recipes).

Unadjusted serum retinol concentration at endline was 0.99 ± 0.03 µmol/L and 0.96 ± 0.02 µmol/L for the yellow cassava and white cassava groups, respectively (**Table 3**). At endline, 17% (*n* = 28) of the children had elevated CRP concentrations (16 in the yellow cassava group and 12 in the white cassava group), and 33% (*n* = 52) were infected with *Plasmodium* parasites (32 in the yellow cassava group and 20 in the white cassava group), with the highest count reaching 57,109 parasites/µL. After adjustment for inflammation, malaria infection accounted for an additional reduction in serum retinol concentrations by 0.03 µmol/L in the entire study population. The occurrence of VAD—adjusted for inflammation—reduced to 8% (*n* = 6 and *n* = 7 in the yellow and white cassava groups, respectively) but did not differ between groups (*P* = 0.22). There was no correlation between RAE intake and adjusted serum retinol concentration (*P* = 0.6).

**TABLE 3:** The treatment effect of consumption of yellow cassava on various outcomes^1^

			Intervention effect	
Outcome/intervention group	*n*	Estimated mean	Crude (95% CI)	Adjusted (95% CI)
Serum retinol concentration, µmol/L				
White cassava group	79	0.96 ± 0.02	Ref	Ref
Yellow cassava group	78	0.99 ± 0.03	0.032 (−0.042, 0.106)^2^	0.06 (0.004, 0.124)^3^
Serum β-carotene concentration, µmol/L				
White cassava group	79	2.51 (1.76, 3.36)^4^	Ref	Ref
Yellow cassava group	78	2.64 (2.10, 3.57)	3.3% (−1.2%, 7.8%)^2^	3.9% (−0.6%, 8.6%)^5^
Serum RBP concentration, µmol/L				
White cassava group	79	0.86 ± 0.03	Ref	Ref
Yellow cassava group	80	0.91 ± 0.03	0.06 (−0.01, 0.13)	0.08 (0.02, 0.14)^6^
Hb concentration, g/L				
White cassava group	79	108.5 ± 9.9	Ref	Ref
Yellow cassava group	80	110.8 ± 10.0	2.32 (−5.45, 0.81)^2^	3.08 (0.38, 5.78)^7^
Lactulose:mannitol ratio				
White cassava group	78	0.021 (0.018, 0.024)	Ref	Ref
Yellow cassava group	77	0.020 (0.017, 0.023)	0.008 (−0.086, 0.103)	0.002 (−0.089, 0.092)^8^

1 Intervention groups were compared using ANCOVA. Hb, hemoglobin; RBP, retinol-binding protein.

2 Only adjusted for RBP stratum.

3 Additionally adjusted for inflammation-adjusted retinol at baseline and for C-reactive protein and malaria status at endline.

4 Values are medians (25th, 75th percentiles).

5 Aditionally adjusted for serum β-carotene at baseline and malaria at endline.

6 Aditionally adjusted for inflammation at baseline, RBP at baseline, and malaria at endline.

7 Aditionally adjusted for Hb at baseline and malaria status at endline.

8 Additionally adjusted for urinary volume.

The intervention resulted in a significant difference in serum retinol concentration between yellow and white cassava groups at endline (Table 3), with a treatment effect of 0.06 µmol/L (95% CI: 0.004, 0.124 µmol/L), after adjustment for randomization stratum, inflammation, and malaria (Table 3). AGP was not associated with serum retinol at baseline (exponential β: 0.03; 95% CI: −0.06, 0.11) or at endline (exponential β: −0.04; 95% CI: −0.12, 0.04). When AGP was included in the model, the effect of the intervention increased to 0.08 µmol/L (*P* = 0.012). A similar treatment effect was found for serum RBP concentration (0.08 µmol/L; 95% CI: 0.02, 0.14 µmol/L), whereas serum β-carotene concentration did not differ between treatment groups (3.9%; 95% CI: −0.6%, 8.6%). No significant treatment effect was found on indicators of gut permeability. However, a significant increase in Hb concentrations was observed in the yellow cassava group (3.08 g/L; 95% CI: 0.38, 5.78 g/L) compared with the white cassava group. At endline, malaria infection alone explained 13% of the variation in Hb concentration, and there was a 14% reduction in Hb in children with malaria (*P* = 0.001). When excluding children with malaria parasites at endline (48 children remaining in the yellow cassava group and 57 in the white cassava group), treatment effects were 0.09 µmol/L (95% CI: 0.02, 0.16 µmol/L) for serum retinol and 3.10 g/L (95% CI: −0.32, 6.52 g/L) for Hb concentrations.

## Discussion

Our results show that daily consumption of biofortified (yellow) cassava modestly prevented a decline in serum retinol concentration in a study population of Nigerian preschoolers after adjusting for inflammation and malaria. The treatment effect was larger for children without malaria parasites in their blood circulation. In addition, we observed a small treatment effect on Hb concentrations. We did not find evidence for an improvement in gut permeability after exposure to the biofortified cassava foods.

The major constraint in the current study was our inability to assess body retinol pools as planned. We excluded a number of potential explanations to be the cause for this, such as *1*) verification of our analytical method by repeating LC-MS/MS analysis in the Lietz’ laboratory at the University of Newcastle upon Tyne (28), *2*) verification of the ^13^C-retinyl acetate dose by HPLC and LC-MS/MS analysis, and *3*) strictly supervised capsule administration to our study participants. In addition, one of the authors took 2 capsules and had her blood taken 4 and 6 d later, showing clear isotope peaks in the plasma. Therefore, in retrospect, the administered dose of 0.4 mg may have been too low and the equilibration period of 14 d too long to be able to still assess the tracer in our study population. Recent studies suggest a shorter equilibration period of 3–6 d to be sufficient for predicting vitamin A stores reliably (37). An additional explanation may be that absorption and retention of the dose were low due to the presence of inflammation in the study population, as shown previously (38).

Much to our surprise, we did find a small but significant treatment effect on the modified primary study outcome: serum retinol concentration. This was unexpected because we did not base our sample size on this outcome. In addition, serum retinol concentration is supposed to be rather irresponsive to intervention when vitamin A status of the population is in the sufficient range, and several colleagues in the field previously failed to show an effect of intervention on this outcome measure (2, 3), whereas others did (4, 39, 40). An important characteristic of studies that do report an effect of intervention on serum retinol concentration is that those studies adjusted their end-of-study values for baseline values. This is justified when the response to treatment depends heavily on starting values, as is the case with serum retinol. Correction for baseline values considerably spikes up the statistical power (41–43). Another important difference from most other intervention studies is that we adjusted for inflammatory markers. The impact of inflammation on serum retinol concentration is quite established (30, 44–46). Helminth, bacterial, and viral infections can trigger inflammatory mechanisms, which consequently lead to a decrease in negative acute phase proteins, such as RBP, responsible for the transportation of retinol from the liver to other body compartments (47, 48). As a result, the mobilization of retinol from the liver to other body compartments may be impaired. Infection-induced inflammation is thus an important confounder of serum retinol concentrations and masks true vitamin A status.

It has been postulated that the presence of malaria parasites lowers serum retinol concentrations independently of inflammatory processes (12, 13). In Nigeria, malaria is endemic, with ∼110 million clinically diagnosed cases of malaria each year (49). Therefore, we attempted to reduce the confounding effect of inflammation by treating all children with deworming medication and antimalarials 2 wk before baseline and endline assessment. However, we still detected *Plasmodium* parasites in 33% of the children at endline. In order to accommodate for this, we included the presence of malaria parasites as a covariate in the model in addition to CRP concentration. We found that the presence of malaria parasites accounted for an additional drop in serum retinol concentrations of 0.03 µmol/L. A similar observation was made in a study conducted in a malaria-endemic region in Burkina Faso, from which the authors concluded that inflammation and malaria appeared to alter serum retinol concentration in a semi-independent manner (45). The possible mechanisms that may explain this phenomenon include *1*) a reduction in RBP synthesis during malaria infection, in a manner independent of the acute phase response; *2*) a parasite-induced impairment in the release of retinol from the liver; and *3*) an increase in the renal excretion of retinol due to malaria infection. Further studies are needed to understand the exact mechanisms responsible for this phenomenon.

Unexpectedly, we did not find any evidence of iron deficiency in the population even at baseline. Possible explanations could be the consumption of foods that are voluntarily fortified or the presence of iron in drinking water, but we currently do not have evidence for either explanation. Previous studies have shown that provision of vitamin A may also increase iron indicators and Hb concentrations (50, 51). This may be explained by the role of vitamin A in stimulating erythropoiesis, thereby mobilizing iron to be incorporated into Hb (52). However, in a malaria endemic region such as Nigeria, the real effect of such interventions may be masked due to the confounding effect of malaria on serum Hb: Malaria infection initiates hemolysis of both parasitized and nonparasitized RBCs, which leads to a depletion of Hb concentrations, thus leading to anemia (53). In the current study, we found a significant effect of yellow cassava on Hb, but only after adjusting for malaria infection.

We also observed that serum β-carotene concentration was very high at baseline (∼2.0 µmol/L) and did not change in response to intervention. This lack of response to treatment may have been due to the 2-wk washout period before endline assessments were conducted. During this period, children returned to consuming nonbiofortified cassava foods, and most of the β-carotene from the intervention available in systemic circulation may have been stored in the liver and/or extrahepatic stores (54). Nevertheless, serum β-carotene concentration was much higher than the 0.17–0.35 µmol/L reported from previous studies conducted in Kenya, Zambia, Bangladesh, and the Philippines (4, 17, 55, 56). For unknown reasons, serum β-carotene concentration seems to be high in Nigerian children, as previously reported by Adelekan et al. (56). This may have to do with the regular intake of high doses of β-carotene from foods prepared with palm oil, which was consumed by >80% of the children at home. We speculate that regular intake of high doses of β-carotene at once may exceed the capacity to convert β-carotene to retinol in the enterocytes, thereby leading to high systemic concentrations of β-carotene. Alternatively, the specific population under study may be genetically predisposed as poor converters of β-carotene. These hypotheses require further investigation.

A major strength of this intervention was that the biofortified meals were prepared using the local recipes available in the community. This resulted in a varied and well-accepted menu for the children. Second, compliance to treatment and study procedures was high due to the preschool that we set up specifically for conduction of the trial. This enabled us to exercise a high level of control over the feeding sessions and all other procedures. Third, we have been able to measure and adjust for major confounders of serum retinol concentrations yielding unbiased treatment effects.

The study was also limited by certain factors. Apart from the necessity to modify the primary outcome measure as outlined previously, it was not possible to control for foods consumed by participants at home outside the intervention meals. However, considering the random allocation to treatment groups, RAE intake at home can be considered to be similar in both groups, which was also reflected in the 24-h dietary recalls, and is therefore not regarded as a confounder of much influence. A second limitation was the low prevalence of VAD in the population, which probably contributed to the small treatment effect. The northern region of Nigeria would have been a better population for this study, based on the high reported prevalence of VAD (7). However, due to logistic and safety reasons, we could not conduct the study in that region. We also could not measure serum zinc as described in the study protocol because there was insufficient serum, which was exhausted during the necessitated repeated analysis. Therefore, we were unable to investigate the role of zinc deficiency as an effect modifier on the study outcomes.

In summary, after adjusting for the effects of inflammation and malaria on serum retinol concentration, a modest treatment effect in serum retinol concentrations was found for daily consumption of biofortified (yellow) cassava. Consumption of yellow cassava can therefore be considered as an efficacious strategy to improve vitamin A concentration in cassava-consuming populations, and especially so in areas and population groups in which vitamin A sufficiency is even more critical.

## Supplementary Material

nqaa290_Supplemental_Files

## Notes

This study was funded by HarvestPlus/International Food Policy Research Institute.

Supplemental Methods and Supplemental Table 1 are available from the “Supplementary data” link in the online posting of the article and from the same link in the online table of contents at https://academic.oup.com/ajcn/.

Data described in the manuscript, code book, and analytical code will be made available upon request, pending application and approval.

Division of Human Nutrition and Health, Wageningen University & Research.

Abbreviations used: AGP, α_1_-acid glycoprotein; BHT, butylhydroquinone; CRP, C-reactive protein; Hb, hemoglobin; RAE, retinol activity equivalent; RBP, retinol-binding protein; RID, retinol isotope dilution; VAD, vitamin A deficiency.
