# Supplementary material for: Daily consumption of pro-vitamin A biofortified (yellow) cassava improves serum retinol concentrations in preschool children in Nigeria: a randomized controlled trial
Source: Am J Clin Nutr. 2020 Nov 12;113(1):221–31. doi: 10.1093/ajcn/nqaa290 (PMC7779233; doi:10.1093/ajcn/nqaa290)
Supplement: nqaa290_Supplemental_Files [file nqaa290_supplemental_files.zip › Supplementary methods_recipes.docx]

**Supplementary Methods: recipes**

Recipe No: Casv_001

Recipe Title: Cassavita II Eba

Number of servings: 200

Serving size: 230 – 250 g

| **Ingredients** | Weight (g) | **Directions/HACCP** |
| --- | --- | --- |
| 1. Garri^1^ | 13,300 | **Critical Control Point (CCP):** Yellow cassava garri should be transported and stored in dark containers Before handling all foods, hands must be washed and clean gloves must be worn.   1. Bring water to boil in 45 – 60 minutes 2. Turn off the heat; add garri gradually to the hot water and stir 3. Turn continuously until a smooth paste consistency is achieved 4. Dish in warmers until the meal is ready to be served |
| 1. Water | 139,900 |  |
| ^1^Garri is roasted cassava (*mannihot spp*) prepared by grinding, grating, dewatering/fermenting and frying raw cassava tubers. The amount of water needed in cooking eba is an estimated average. The exact amount of water needed to produce the same consistency may differ slightly. | | |

Recipe No: Casv_002

Recipe Title: Cassavita II Boiled Meat

Number of servings 200

Serving size: 12 g each (cooked)

| **Ingredients** | Weight  (g) | **Directions/HACCP** |
| --- | --- | --- |
| 1. Raw lean meat | 4,500-5,000  (200 pieces;  ~23g/piece) | **Critical Control Point (CCP):** Before handling all foods, hands must be washed and clean gloves must be worn.   1. Wash lean meat in clean water 2. Put meat in a hot pot placed on cooker 3. Slice onions into meat using a knife 4. Add water 5. Add salt to taste 6. Stir using a cooking spoon 7. Cover pot to boil for 40minutes till meat is soft 8. Turn off the heat and separate meat from stock |
| 1. Peeled onions *(Allium Cepa)* | 300-350 |  |
| 1. Water | 3,650 |  |
| 1. Salt |  |  |
|  | | |

Recipe No: Casv_003

Recipe Title: Cassavita II Stew

Number of servings 200

Serving size: 35 g

| **Ingredients** | Weight  (g) | **Directions/HACCP** |
| --- | --- | --- |
| 1. Tomatoes | 3,000 | **Critical Control Point (CCP):** Before handling all foods, hands must be washed and clean gloves must be worn.   1. Blend all ingredients (apart from salt and vegetable oil) using an industrial pepper grinder. 2. Pour blended pepper in a pot containing meat stock (which has been put on fire) 3. Add salt to taste 4. Add little water (if required) 5. Add vegetable oil and stir 6. Boil for 25 minutes 7. Turn off heat |
| 1. Long pepper *(Capsicum spp)* | 1,008 |  |
| 1. Chili pepper *(Capsicum spp)* | 76 |  |
| 1. Sweet pepper *(Capsicum annuum)* | 228 |  |
| 1. Peeled onions *(Allium cepa)* | 960 |  |
| 1. Vegetable oil (unfortified) | 750 |  |
| 1. Meat stock^1^ | ?? |  |
| 1. Water | 4,500 |  |
| 1. Salt to taste |  |  |

^1^Meat stock was the liquid obtained after boiling beef in water, see recipe no Casv_002.

Recipe No: Casv_004

Recipe Title: Cassavita II Okra Vegetable Soup

Number of servings 200

Serving size: 35 g

| **Ingredients** | Weight  (g) | **Directions/HACCP** |
| --- | --- | --- |
| 1. Okra fruits *(Abelmoschus esculentus)* | 3,500 | **Critical Control Point (CCP):** Before handling all foods, hands must be washed and clean gloves must be worn.   1. Wash okra fruits free from dirt 2. Grate using an okra grater 3. Bring water to boil 4. Add the grated okra fruits and the locust beans 5. Add salt to taste 6. Bring to boil again and stir continuously for 15-20 minutes till soft and a slimy/drawing consistency is attained. |
| 1. Water | 5,100 |  |
| 1. Locust beans (*Parkia biglobosa*) | 210 |  |
| 1. Salt to taste |  |  |

Recipe No: Casv_005

Recipe Title: Cassavita II *Ewedu* Vegetable Soup

Number of servings 200

Serving size: 20 g

| **Ingredients** | Weight  (g) | **Directions/HACCP** |
| --- | --- | --- |
| 1. Ewedu leaves *(chocorus olitorus)* | 1350 | **Critical Control Point (CCP):** Before handling all foods, hands must be washed and clean gloves must be worn.   1. Pluck the leaves from the stem 2. Wash leaves thoroughly with water 3. Bring water to boil and pour in the leaves 4. Add locust beans 5. Add salt 6. Stir continuously and bring again to boil (15-20 mins) until the leaves become soft 7. Blend (using a kitchen blender) to form a puree |
| 1. Water | 8,550 |  |
| 1. Locust beans (*Parkia biglobosa*) | 156 |  |
| 1. Salt to taste |  |  |

Recipe No: Casv_006Recipe Title: Cassavita II Moinmoin

Number of servings 200

Serving size: 127 g each (uncooked)

| **Ingredients** | Weight (g/mls) | **Directions/HACCP** |
| --- | --- | --- |
| 1. Yellow cassava roots (*peeled and cubed, yielding 12,423 g of cassava mash*) | 14,250 | **Critical Control Point (CCP):** Before handling all foods, hands must be washed and clean gloves must be worn.   1. Peel the cassava roots; wash them in clean water and cut into cubes using a knife (manually) 2. Weigh cubed cassava 3. Grind cubed cassava into a smooth slurry, using an industrial grinder 4. Drape a chiffon cloth *(for white cassava)* and cloth bag (*for yellow cassava)* over a big bowl. 5. Pour the mixture into the cloth in batches to separate the liquid from the mash (dewatering). 6. Weigh required cowpea slurry 7. Blend peppers and onions using a blender or grinder 8. Homogenize cassava mash, with the pepper/onion mixture, cowpea slurry, and vegetable oil. Add salt to taste. 9. Portion this mixture into moinmoin leafs and mold in way that it avoids the mixture from pouring or leaking out 10. Arrange these portions into a pot of boiling water^2^. Cover tightly and allow steaming for 45 minutes to 1 hour 11. Turn off the heat and serve |
| 1. White cassava roots (*peeled and cubed, yielding 12,423 g of cassava mash*) | 14,250 |  |
| 1. Cowpeas for slurry^1^ | 3,106 |  |
| 1. Long pepper *(Capiscum spp)* | 724 |  |
| 1. Chili pepper *(Capiscum spp)* | 91g |  |
| 1. Peeled onions *(Allium Cepa)* | 1,354 |  |
| 1. Water to grind cassava | 7,000 |  |
| 1. Water to grind pepper | 500 |  |
| 1. Vegetable oil (unfortified) | 2,081 |  |
| 1. Salt to taste |  |  |
|  | |  |
| ^1^Preparation of bean slurry: measure dry beans, put in water and remove the coat almost immediately, soak in water for about 10 minutes, decant, grind in an industrial grinder and add water gradually until well blended into slurry  ^2^The water in the pot should be minimal, such that it doesn’t cover the portions of the food. Add water to the inner base of the pot if needed at intervals of steaming to avoid burning of the moinmoin | | |
