# Supplementary material for: Daily consumption of pro-vitamin A biofortified (yellow) cassava improves serum retinol concentrations in preschool children in Nigeria: a randomized controlled trial
Source: Am J Clin Nutr. 2020 Nov 12;113(1):221–31. doi: 10.1093/ajcn/nqaa290 (PMC7779233; doi:10.1093/ajcn/nqaa290)
Supplement: nqaa290_Supplemental_Files [file nqaa290_supplemental_files.zip › Supplementary Table 1.docx]

**Supplementary Table 1**: β-Carotene Content of Intervention Food Samples

| Food Sample | Trans-β-Carotene  µg/100 g | | Cis-β-Carotene  µg/100 g | | Total β-Carotene  µg/100 g | Total β-Carotene  µg/100 g | |
| --- | --- | --- | --- | --- | --- | --- | --- |
|  | Mean | SD | Mean | SD | Mean | Average of means | SD |
| Ewedu soup 1 | 783.2 | 2.1 | 690.7 | 8.4 | 1474 | 1231 | 344 |
| Ewedu soup 2 | 768.0 | 37.5 | 220.0 | 10.1 | 988.0 |  |  |
| Okra soup 1 | 319.0 | 7.0 | 136.4 | 7.7 | 455.5 | 409.6 | 64.8 |
| Okra soup 2 | 268.5 | 12.8 | 95.3 | 8.0 | 363.8 |  |  |
| White cassava eba 1 | 12.6 | 0.9 | 5.0 | 5.0 | 17.57 | 13.99 | 12.6 |
| White cassava eba 2 | 14.4 | 2.6 | 10.0 | 4.9 | 24.40 |  |  |
| White cassava eba 3 | 0.0 | 0.0 | 0.0 | 0.0 | 0.00 |  |  |
| White cassava garri 1 | 20.5 | 7.3 | 52.1 | 29.4 | 72.60 | 50.66 | 19.0 |
| White cassava garri 2 | 18.8 | 4.1 | 20.5 | 5.7 | 39.27 |  |  |
| White cassava garri 3 | 22.5 | 1.3 | 17.6 | 2.1 | 40.10 |  |  |
| White cassava moinmoin 1 | 88.7 | 4.3 | 30.1 | 8.5 | 118.8 | 136.7 | 20.5 |
| White cassava moinmoin 2 | 113.2 | 0.3 | 45.8 | 5.2 | 159.1 |  |  |
| White cassava moinmoin 3 | 88.0 | 3.9 | 44.3 | 2.1 | 132.3 |  |  |
| Yellow cassava eba 1 | 168.7 | 6.6 | 167.0 | 7.7 | 335.7 | 318.2 | 16.0 |
| Yellow cassava eba 2 | 146.2 | 1.1 | 168.3 | 8.2 | 314.6 |  |  |
| Yellow cassava eba 3 | 122.1 | 3.9 | 182.2 | 16.7 | 304.4 |  |  |
| Yellow cassava garri 1 | 388.1 | 7.8 | 465.4 | 0.9 | 853.5 | 973.6 | 174 |
| Yellow cassava garri 2 | 392.3 | 4.3 | 501.9 | 0.3 | 894.2 |  |  |
| Yellow cassava garri 3 | 499.4 | 19.3 | 673.5 | 11.6 | 1173 |  |  |
| Yellow cassava moinmoin 1 | 106.1 | 5.5 | 60.4 | 6.5 | 166.5 | 286.2 | 108 |
| Yellow cassava moinmoin 2 | 175.9 | 8.2 | 141.1 | 21.4 | 317.0 |  |  |
| Yellow cassava moinmoin 3 | 234.6 | 4.3 | 140.3 | 12.7 | 375.0 |  |  |
